# Hepatoprotective Property of Oral Silymarin is Comparable to N-Acetyl Cysteine in Acetaminophen Poisoning

**DOI:** 10.4021/gr463e

**Published:** 2012-09-20

**Authors:** Amir Mohammad Kazemifar, Ali Akbar Hajaghamohammadi, Rasoul Samimi, Zohreh Alavi, Esmail Abbasi, Marjan Nasiri Asl

**Affiliations:** a Internal Medicine Department, Qazvin’s University of Medical Sciences, Qazvin, Iran; b Iranian Legal Medicine Organization, Tehran, Iran; c Qazvin’s University of Medical Sciences, Qazvin, Iran

**Keywords:** Acetaminophen, Poisoning, Hepatotoxicity, N-Acetyl Cysteine, Silymarin

## Abstract

**Background:**

N-Acetyl Cysteine (NAC) is usually used as antidote for prevention of acetaminophen-induced hepatotoxicity. In present study we have evaluated efficacy of oral silymarin in its prevention in rats intoxicated with lethal dose of acetaminophen.

**Methods:**

A total of 50 Male Sprague-Dawley rats were randomly divided into five groups. The first group received only vehicle of acetaminophen and served as control. The second group was given 800 mg/kg acetaminophen by gavage with an orogastric canula. The third, fourth and fifth groups were given 300 mg/kg NAC and 150 and 300 mg/kg silymarin respectively. Analysis of serum AST, ALT, and ALP and liver histopathology were employed for assessment of hepatotoxicity.

**Results:**

Mean serum ALT levels were significantly increased in the APAP group rats. The mean serum ALT levels returned to normal in both NAC treated and silymarin treated groups. Silymarin (150 mg/kg) had prevented hepatocytes necrosis similar to NAC. No severe hepatotoxicity were seen in groups 3 and 4; while it is seen in 70% of animals in group 2.

**Conclusion:**

We found that a single dose of orally administered silymarin (150 mg/kg) significantly attenuated acetaminophen-induced liver damage in rat. Oral silymarin can be used in these patients instead of NAC.

## Introduction

Drug-induced liver injury is often life-threatening. It is a major reason for withdrawal of drugs from the market and cessation of new drug development [1].

Acetaminophen is a commonly used analgesic and antipyretic agent for relieving mild and moderate pain. It is available as an over-the-counter medication. However, acetaminophen-induced hepatotoxicity, caused by deliberate or accidental overdose, is now the most frequent cause of fulminant liver failure in the Western world and has a mortality rate of 90% [2].

Recent epidemiological studies have shown that the hospitalization rate due accidental or intentional APAP overdose is estimated to be over than 26,000 cases per year, being considered as the major cause of liver failure, hepatic transplant and often used in suicides attempts [3].

The toxicity is initiated by cytochrome P450 (CYP) metabolism into N-acetyl-p-benzoquinone imine (NAPQI), and the high reactivity of NAPQI with sulfhydryl groups results in depletion of reduced glutathione (GSH) in hepatocytes, followed by covalent binding to intracellular proteins [1].

The acetaminophen risk analysis nomogram is used to predict the risk of hepatotoxicity based on a single plasma acetaminophen concentration (PAC) measured between 4 and 24 h after an acetaminophen overdose [4].

N-Acetyl Cysteine (NAC) is usually used as antidote for prevention of acetaminophen-induced hepatotoxicity. It can be used orally or parenterally [5]. Regardless of route of administration its use may be associated with some adverse reactions [6].

Experimentation with other possible drugs that may be effective as antidote for acetaminophen-induced hepatotoxicity has been done; which have suggested some drugs such as Fumaria [7], Galic acid [8], Taurine [9], and silymarin [10].

In present study we have evaluated efficacy of oral silymarin in prevention of acetaminophen-induced hepatotoxicity with lethal dose of acetaminophen in rat.

## Material and Methods

### Chemicals

Acetaminophen was from Sigma Chemical Co., USA. Acetaminophen was dissolved in normal saline before use. NAC with trade name EXI-NACE were from Exir pharmaceutical co., Iran in form of 10 Ml vial with concentration of 200 mg/mL.

Silymarin with trade name Livergol were from Goldaru pharmaceutical co., Iran in form of 140 mg tablet. Both drugs were purchased from a pharmacy. They were dissolved in normal saline too.

### Animals

Male Sprague-Dawley rats, weighing 230 ± 10 gr. were obtained from Razi research institute, Karaj city, Iran. The animals were kept in standard conditions (22 ± 2 °C, 45 ± 5% humidity and 12 h light/dark cycle). They were supplied with standard laboratory diet and water ad libitum, and left to acclimatize for 2 weeks before the experiments. Rats were allowed free access to water but not food for 6 h before the experiment.

### Experimental design

The rats were randomly divided into five equal groups (n = 10 in each group).

The first group received a single oral dose of normal saline (vehicle of acetaminophen) and served as control.

Hepatotoxicity was induced in animals of the second group by acetaminophen given at a single oral dose of 800 mg/kg by gavage with an orogastric canula.

The third group of animals were received NAC simultaneous with acetaminophen same as second group, given at a single oral dose of 300 mg/kg by gavage.

The fourth and fifth groups of animals were received silymarin concomitant with acetaminophen equivalent to second group, given at a single oral dose of 150 and 300 mg/kg by gavage respectively.

The experimental protocol was approved by the Local Animal Care Committee at Qazvin’s university of medical sciences, Qazvin, Iran. The experimental procedures were carried out in accordance with international guidelines for the care and use of laboratory animals.

After 72 hours, the rats were anesthetized by xylazine 2% and Ketamine 10%; then 5 mL from their blood were taken from the heart. Afterward, the abdomen was opened, and the livers were removed and cleaned. Liver tissue samples were stored in 10% formalin solution for histopathology analysis.

### Assessing hepatic injury

The collected blood samples from all animals via cardiac puncture were allowed to clot. Their serum was removed by centrifugation at 1000 × g for 10 min at room temperature. All serum samples were sterile and haemolysis-free. They were processed to determine the enzymatic activities of aspartate aminotransferase (AST), alanine aminotransferase (ALT), and alkaline phosphatase (ALP) with a spectrophotometric technique by the Olympus AU-2700 auto analyzer using commercial kits (Pars Azmoon, Iran) and presented as IU/L.

For liver histopathology analysis, we processed midsections of the left lobes of the liver for light microscopy. The specimens were processed as standard methods, then were stained with hematoxylin-eosin (H&E) and trichrome. The liver tissue sections were independently examined and scored by one of the authors; while the examiner was unaware of the group to which the specimen belonged.

The degree of necrosis was expressed as the mean of 10 high power fields (HPFs), chosen at random and classified on a scale of 0 - 5 (no hepatocyte necrosis, 0; necrosis in few hepatocytes, 1; necrosis in more than 10% but less than 24% of hepatocytes, 2; necrosis in more than 25% but less than 39% of hepatocytes, 3; necrosis in more than 40% but less than 49% of hepatocytes, 4; and necrosis in more than 50% of hepatocytes, 5) as described and used by Silva [11].

### Statistical analysis

We used SPSS 16.0 statistical package to perform all statistical analyses. One-way ANOVA and chi-square tests were used to test differences between the groups.

The results were expressed as mean ± standard deviation (SD). A probability level of < 0.05 was considered statistically significant

## Results

### AST, ALT and alkaline phosphatase levels

Table 1 shows the results of the AST, ALT and Alkaline Phosphatase (ALP) measurements.

**Table 1:** Levels of Serum Liver Enzymes in Studied Groups (Mean ± Standard Deviation)

	ALT level (IU/L)	AST level (IU/L)	ALP level (IU/L)
Group 1	28.82 ± 10.37	153.36 ± 23.70	163.18 ± 34.83
Group 2	93.89 ± 89.43	194 ± 71.45	266.67 ± 90.92
Group 3	26.18 ± 8.13	102 ± 45.27	247 ± 64.17
Group 4	31.89 ± 10.46	157.44 ± 70.75	255.33 ± 134.0
Group 5	40 ± 11.81	169.27 ± 38.42	256.82 ± 144.14
F	5.04	4.42	1.85
Level of significant difference between groups^*^	0.002	0.004	0.134

^*^: as were tested by one-way ANOVA.

Mean serum ALT levels were significantly increased in the APAP group rats when compared to normal rats. The mean serum ALT levels returned to normal in both NAC treated and silymarin treated groups (Table 1).

Post HOC tests include Tukey HSD and Dunnett showed that AST levels in group 3 (NAC treated) and 4 (silymarin treated, 150 mg/kg) have not statistically significant difference; while both are statistically different from the level in group 2 (acetaminophen intoxicated treated). Also mean AST and ALP levels are not statistically significant in group 3 and 4.

### Liver injury

The results of histopathologic grading were shown in Table 2. The increased serum

**Table 2:** Histopathologic Grading of Liver Necrosis in Studied Groups (Mean ± Standard Deviation)

	Histopathologic grading
Group 2	3.44 ± 1.130
Group 3	1.30 ± 0.483
Group 4	1.38 ± 0.744
Group 5	3.67 ± 1.732

ALT (indicative of necrosis) activities in APAP-induced rats agreed with the histopathological liver injury. Silymarin (150 mg/kg) had reduced hepatocytes necrosis similar to NAC. If grades 3 and higher was regarded as severe hepatotoxicity, no severe hepatotoxicity were seen in groups 3 and 4; while it is seen in 70% of animals in group 2 and 5.

## Discussion

We found that a single dose of orally administered silymarin (150 mg/kg) significantly attenuated acetaminophen-induced liver damage in rat. If the dose is increased to 300 mg/kg, it did not protect the liver from acetaminophen-induced hepatic damage.

Recently, there has been wide interest in the role of oxidative stress in the pathogenesis and progression of liver diseases, as well as in several drug-induced hepatotoxicity models, particularly in APAP induced liver injury [12, 13]. As the deliberated use of this drug is still increasing, the research for new compounds that act against oxidative stress in intoxicated hepatocytes without causing any damage to these cells and other organs is relevant.

The extracts of the flowers and leaves of *silybum marianum* have been used for centuries to treat liver disorders. One of the important issues about the plant is that it may be accepted as a safe herbal product with no health hazard or significant side effect [14]. Its anti-oxidant property was demonstrated in previous studies [15]. The most extensively studied and disseminated property of silymarin is its hepatoprotective activity. Several clinical studies have been performed to evaluate the efficacy of silymarin to treat a range of liver and gallbladder disorders such as acute and chronic hepatitis, cirrhosis and toxin-induced hepatitis [16].

It is believed to act as antioxidant, anti-inflammatory and anti-fibrotic agents. The anti inflammatory effect seems to involve blocking the activation of intrahepatic Nuclear Factor kappa B (NF-κB) and consequent diminution of Tumor Necrosis Factor-alpha (TNF-α), Interferon (IFNg), IL-2 and inducible Nitric Oxide Synthase (iNOS). The ability to act as cellular antioxidants, on the other hand, has been attributed to the many beneficial effects of silymarin. These effects were associated with decreased membrane lipid peroxidation, reduced free-radical release and restoration in the GSH levels [16].

Despite a long history of its use and the large number of people who consume this substance, no conclusive data on its clinical efficacy can be identified. In fact, only a few well designed clinical trials have been performed. Most studies have been conducted using silymarin and with inclusion of patients with alcoholic or viral cirrhosis. In field of toxicology, administration of silymarin after poisoning produced by the mushroom Amanita phalloides (death cap) seems to be an effective measure to prevent severe liver damage [17].

Compos and his coworkers have showed effects of silymarin in protection of rat liver against Glutathione depletion after acetaminophen toxicity [18]. On the other hand, Muriel and his colleagues have claimed that silymarin can not prevent Glutathione depletion following acetaminophen overdose and it protects liver by reducing lipid peroxidation. They had given silymarin to rats 24 hours before Acetaminophen poisoning [19]. Avizeh and his collaborators have examined role of silymarin in acetaminophen toxicity in cats that have distinct sensitivity to the drug, presenting with methemoglobinemia [20]. They concluded that silymarin can protect liver tissue against oxidative stress in cats with APAP intoxication.

Current study was conducted to evaluate efficacy of silymarin in prevention of APAP toxicity in rat with some distinct aspects in arrangement of the study: 1) APAP was given orally to reproduce the true condition of the poisoning; 2) APAP was given in lethal dose (800 mg/kg); not toxic dose; 3) Silymarin was given orally and simultaneous with APAP; 4) Assessment of hepatic injury was done 72 hours after the poisoning; 5) Two suggested doses of silymarin were evaluated in the study.

Results of present study showed that silymarin can substitute for NAC in treatment of APAP poisoning in an animal model. Design of similar study in human subjects can help to confirm the conclusion; if possible. Till that time, if NAC is not accessible; especially in primary care centers of distant areas and prehospital settings, oral silymarin can be used in patients with APAP poisoning instead.

Additionally silymarin may be used for production of a safe form of acetaminophen composed from acetaminophen plus silymarin; consequently when accidental or intentional overdose take places, the drug will be ingested with its antidote.
